# Effects of biochar addition on nitrous oxide emission during soil freeze–thaw cycles

**DOI:** 10.3389/fmicb.2022.1033210

**Published:** 2022-10-18

**Authors:** Zhihan Yang, Ruihuan She, Lanfang Hu, Yongxiang Yu, Huaiying Yao

**Affiliations:** ^1^Research Center for Environmental Ecology and Engineering, School of Environmental Ecology and Biological Engineering, Wuhan Institute of Technology, Hubei, China; ^2^Key Laboratory of Urban Environment and Health, Ningbo Observation and Research Station, Institute of Urban Environment, Chinese Academy of Sciences, Fujian, China; ^3^Zhejiang Key Laboratory of Urban Environmental Processes and Pollution Control, CAS Haixi Industrial Technology Innovation Center in Beilun, Zhejiang, China

**Keywords:** freezing–thawing, soil moisture, biochar, nitrous oxide, paddy soil

## Abstract

Biochar applied to soil can reduce nitrous oxide (N_2_O) emissions produced by freeze–thaw processes. Nonetheless, how biochar modification affects N_2_O emissions during freeze–thaw cycles is not completely clear. In our research, during freeze–thaw cycles, microcosm experiments were conducted to investigate the effects of maize straw biochar (MB) or rice straw biochar (RB) addition on soil N_2_O emissions under different water conditions. The N_2_O emissions peaked at the initial stage of thawing in all the soils, and the total N_2_O emissions were considerably greater in the flooded soils than in the nonflooded soils. Compared with the soils without biochar addition, RB and MB amendments inhibited N_2_O emissions by 69 and 67%, respectively. Moreover, after biochar addition, the abundance of AOB *amoA* genes decreased by 9–13%. Biochar addition significantly decreased the content of microbial biomass nitrogen (MBN) in flooded soil during thawing, which was significantly correlated with N_2_O emissions and nitrification and denitrification communities. The PLS-PM further revealed that biochar can inhibit the production and emission of soil N_2_O by reducing soil MBN during soil thawing. In addition, soil moisture directly significantly affects N_2_O emissions and indirectly affects N_2_O emissions through its influence on soil physicochemical properties. Our results revealed the important function of biochar in decreasing the emission of N_2_O in flooded soil during freeze–thaw cycles.

## Introduction

Freeze–thaw alternation, as a familiar natural phenomenon, affects more than 70% of the land area in cold regions and high latitudes (Mellander et al., 2007). Global climate warming has exacerbated the intensity and frequency of freeze–thaw cycles, leading to an increase in nitrous oxide (N_2_O) release in soil. On a 100-year timescale, the global warming potential of N_2_O, a significant greenhouse gas, is 298 times higher than that of carbon dioxide (CO_2_; Gao et al., 2015; Pelster et al., 2019). Soil-sourced N_2_O emissions account for 60% of total N_2_O emissions, and up to 70% of the annual N_2_O flux could be emitted in temperate regions during the freeze–thaw period (Yao et al., 2010). In a laboratory experiment, Gao et al. (2015) found that the total N_2_O emissions from peatland and meadow soils in the Qinghai-Tibet Plateau sharply increased by 5.8 and 3.9 times after freezing and thawing, respectively. In forest fields, Peng et al. (2019) conducted high-frequency monitoring of N_2_O emissions on Changbai Mountain and found that the average daily N_2_O emissions rate in the freeze–thaw period was up to 2618.3 μg N m^−2^d^−1^, and the cumulative emissions in the freeze–thaw period accounted for approximately 58% of the annual emissions. Based on a model, Wagner-Riddle et al. (2017) predicted that farmland soils undergoing seasonal freeze–thaw contributed approximately 1.07 ± 0.59 Tg of N_2_O per year, which is considered the largest anthropogenic source of N_2_O to date. The enhanced metabolism of substrate microorganisms accumulated during thawing is considered the most likely cause of the high risk of N_2_O emission (Kim et al., 2012). Ice films are formed on the surface of soil granules in the process of soil freezing, leading to an anoxic environment and promoting microbial denitrification, hence generating N_2_O. Nevertheless, the presence of ice film also inhibited the release of N_2_O. While the soil commenced to melt, all captured N_2_O was quickly released into the atmosphere (Goldberg et al., 2010).

For the past few years, numerous studies have reported that biochar applied to soils affects the emission of N_2_O (Purakayastha et al., 2019; Ahmad et al., 2021; Zhang et al., 2022a). Although biochar contains α-pinene and ethylene, volatile organic compounds called nitrification inhibitors (Spokas et al., 2011; Taghizadeh-Toosi et al., 2011), these influences differ across biochar and soil types and environmental conditions. For example, Bruun et al. (2011) and Clough et al. (2010) discovered that biochar combined with bovine urine or anaerobically digested slurry enhanced soil N_2_O emissions. However, it was also asserted that biochar addition did not influence soil N_2_O emissions in subtropical grasslands (Scheer et al., 2011). Until now, the impact of biochar on nitrification and denitrification related to N_2_O formation remained unclear. For example, Case et al. (2015) found that biochar addition accelerated nitrification and soil N mineralization to produce N_2_O, but this practice decreased the total emissions of N_2_O by 91% by inhibiting denitrification, which was probably due to the different properties of biochar. Therefore, it is important to investigate the effects of different types of biochar on N_2_O emissions during thawing.

As an important environmental factor related to oxygen availability in soil, moisture is closely coupled with biogeochemical cycles (Song et al., 2020). Soil moisture directly affects soil microorganism activity and indirectly regulates nitrification and denitrification processes by affecting soil substrate availability and oxygen diffusion capacity (Chen et al., 2018). Chen et al. (2018) found that soil moisture was positively related to N_2_O flux in the nongrowing period and explained approximately 32% of the change in N_2_O flux. Initial soil moisture conditions also affect the dynamics of N transformation during freeze–thaw (Stres et al., 2008). Anaerobic or anoxic microenvironments were generally beneficial for denitrifiers to produce N_2_O. With the gradual increase in water content, the degree of damage caused by water molecules to soil aggregates becomes more pronounced during freezing. Accordingly, the higher amount of active organic carbon released by the aggregates probably promotes the metabolic activity of heterotrophic microorganisms, including denitrifying microorganisms (Song et al., 2020). In this experiment, maize straw biochar and rice straw biochar were selected to explore the regulatory effect of different biochars on N_2_O emissions during the soil freeze–thaw cycles under different water conditions. We hypothesized that biochar addition can reduce N_2_O emissions by decreasing the abundance of functional genes related to denitrification.

## Materials and methods

### Soil analysis

The studied soil was gathered from a typical paddy field that has a long history of rice cultivation in Shuguang Village (123°58′31″ E, 47°23′15” N), Qiqihar City, Heilongjiang Province, China. Influenced by the mid-temperate continental monsoon climate in this area, the average annual temperature was 3.2°C (the monthly average temperature ranged from −20.5°C in January to 22.2°C in July), and the average annual precipitation was 415 mm. The topsoil normally begins to frost around October and thaws in March of the following year due to rising temperatures (Song et al., 2008). We collected surface-layer soil (0–20 cm) after the thawing period in March 2019. Part of the soil samples were air-dried and then sieved through a 2-mm mesh to remove observable stones and roots for physicochemical analysis. The other part of the soil sample was stored at 4°C for subsequent incubation experiments. The soil texture was silty loam. The pH, total nitrogen and total carbon contents of the soil were 6.4, 0.22, and 2.37%, respectively. The basic chemical and physical properties of the soil are listed in Table 1.

**Table 1:** Physicochemical properties of soil samples and biochar.

	C (%)	N (%)	C/N ratio	pH	Soil texture		
					Sand (%)	Silt (%)	Clay (%)
Soil	2.37	0.22	10.4	6.4	38	26	36
MB	75.3	1.35	55.9	8.5	–	–	–
RB	46.5	0.76	60.9	10.4	–	–	–

MB, maize straw biochar; RB, rice straw biochar.

### Experimental design

**Figure 1:**
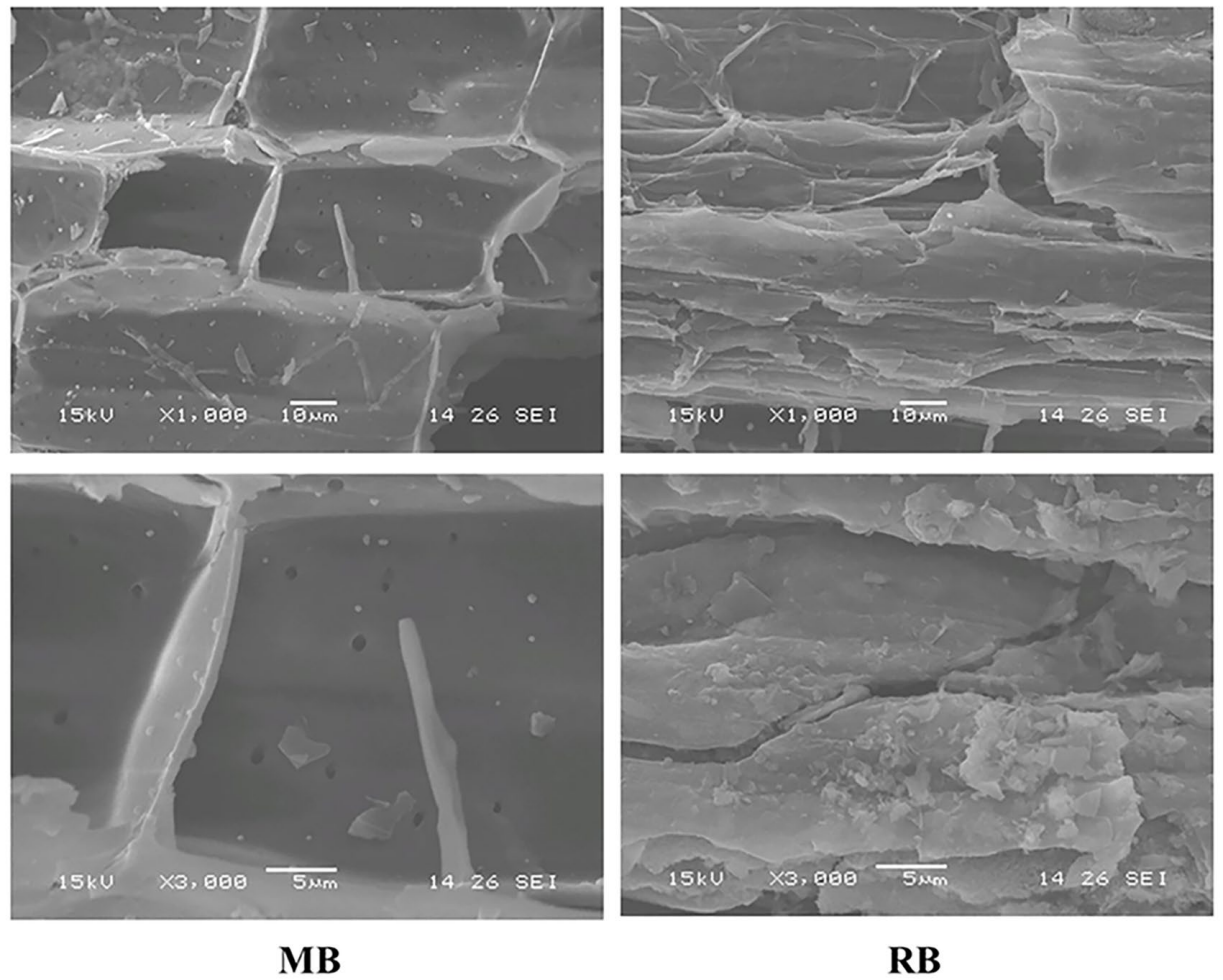
Surface morphology and structural characteristics of biochar observed by scanning electron microscopy (SEM; MB: maize straw biochar; RB: rice straw biochar).

Soil samples of 15.0 g (dry weight) were accurately weighed in a 120-ml brown serum flask, and the soil water content was adjusted to 60% water holding capacity (WHC), 100% WHC, and flooding with a 1-cm water layer thickness. Maize straw biochar (MB) and rice straw biochar (RB) with a mass concentration of 2% were added before incubation. The basic physicochemical properties of the biochar used in this study are listed in Table 1. The surface morphology and structural characteristics of biochar were observed by scanning electron microscopy (SEM; Figure 1). All treatments were precultured at 25°C for 7 days in the dark (the culture vessel was ventilated, and water loss was replenished) to fully recover the microbial activity in the soil. After preculture, the soils were frozen at −20°C for 7 days to simulate winter freezing, and then the temperature was set to 4°C for 12 days to simulate thawing.

### Gas sampling and analysis

Nitrous oxide emissions were measured within 12 days after thawing. A gas chromatograph with an electron capture detector (ECD) was used to analyze the concentration of N_2_O produced by the soil. Then, the total N_2_O emissions of the entire culture period were calculated by the daily emission rate described by Yu et al. (2022).

### Soil mineral nitrogen measurement

The cultured fresh soil samples on the first day after thawing were weighed to approximately 3 g, 1 mol/L KCl solution was added at a ratio of 1:10 (w:v), and the sample was fully mixed. The samples were placed on a horizontal oscillator to fully oscillate for 30 min (180 r/min). After shaking, the filtrate was collected by centrifugation at 3,000 r/min. Finally, a flow analyzer determined the contents of nitrate (NO_3_^−^) and ammonium (NH_4_^+^; Zhang et al., 2022b).

### Soil dissolved organic carbon and microbial biomass nitrogen measurements

The microbial biomass nitrogen (MBN) contents in the soil were determined by potassium sulfate extraction and chloroform fumigation (Jenkinson et al., 2004). Approximately 3 g of fresh soil on the first day after thawing was weighed and placed in a clean centrifuge tube, and 0.5 ml of ethanol-free chloroform was added and fully mixed. The soils were placed at 25°C for sealed culture in the dark for 24 h, and 10 ml potassium sulfate solution with a concentration of 0.5 mol/l was added. After shaking extraction for 30 min (180 r/min) on the vibrating machine, centrifugal filtration was carried out, and finally, the measurement was carried out on the machine. The control group was extracted without fumigation and determined by potassium sulfate solution. The soil dissolved organic carbon and nitrogen contents were analyzed by a total organic carbon analyzer (MULTI-N/C 2100S, Analytik Jena, Jena, Germany). The organic carbon concentration of the unfumigated sample group is the dissolved organic carbon (DOC) content of the soil sample. The MBN contents were obtained from the difference in total nitrogen (TN) between the fumigated and unfumigated soils multiplied by the corresponding conversion coefficient (kEN = 0.45; Jenkinson et al., 2004).

### DNA extraction and quantitative PCR analysis

DNA was extracted from 0.5 g freeze-dried soil using the FastDNA® for Soil Kit (MP Biomedicals, California, United States) strictly according to the manufacturer’s instructions. The DNA of the soil samples before freezing and on the first and seventh days of thawing was extracted. Quantitative analysis of functional genes is based on extracted DNA as templates at the gene level. Thus, DNA was used as a template for quantitative analysis of nitrification [ammonia-oxidizing archaea (AOA) *amoA*, ammonia-oxidizing bacteria (AOB) *amoA*] and denitrification (*nirS*, *nirK*, and *nosZ*) functional genes (Zhang et al., 2021). The detailed amplification system, conditions and primers are shown in Table 2.

**Table 2:** Primers for nitrifier (AOA and AOB *amoA*) and denitrifier (*nirK*, *nirS* and *nosZ*) genes and their thermal cycling conditions for qPCR.

Target genes	Primer	Sequence (5′–3′)	Product length (bp)	Amplification condition	References
AOA *amoA*	*CrenamoA*23f	ATGGTCTGGCTWAGACG	635	95°C 15 s; 53°C 45 s; 72°C 45 s; 83°C 15 s; 45 cycles	Francis et al. (2005)
	*CrenamoA*616r	GCCATCCATCTGTATGTCCA			
AOB *amoA*	*amoA*1F	GGGGTTTCTACTGGTGGTCCC	491	95°C 15 s; 54°C 40 s; 72°C 45 s; 84°C 15 s; 40 cycles	Francis et al. (2005)
	*amoA*2R	CTCKGSAAAGCCTTCTTC			
*nirS*	*nirS*-cd3aF	GTSAACGTSAAGGARACSGG	425	95°C 15 s; 50°C 45 s; 72°C 45 s; 88°C 15 s; 50 cycles	Throback et al. (2004)
	*nirS*-R3cd	GASTTCGGRTGSGTCTTGA			
*nirK*	*F1aCu*	ATCATGGTSCTGCCGCG	514	95°C 10 s; 53°C 45 s; 72°C 45 s; 86°C 15 s; 45 cycles	Throback et al. (2004)
	*R3Cu*	GCCTCGATCAGRTTGTGGTT			
*nosZ*	*nosZ*-F	CGYTGTTCMTCGACAGCCAG	453	95°C 15 s; 50°C 30 s; 72°C 30 s; 83°C 15 s; 55 cycles	Scala and Kerkhof (1998)
	*nosZ*-1662R	CGSACCTTSTTGCCSTYGCG			

Quantitative PCR (qPCR) was carried out for analysis using the LightCycler® 480II system (Roche Diagnostics, Basel, Switzerland). The amplification system contained 10 μl Absolute SYBER Fluorescein Mix (Thermo Scientific, New York, United States), 0.5 μl primer, 7 μl nuclease-free water, and 2 μl 10-fold diluted DNA stock as a template (6–26 ng). Negative controls were replaced with the same amount of nuclease-free water. The functional gene plasmids related to bacterial nitrification and denitrification were extracted by DNA template, and the plasmids were continuously diluted 10 times to form a standard curve for gene quantitative analysis (Yin et al., 2019).

### Statistical analysis

The software used for data processing and analysis mainly included Excel 2016, OriginPro 2018, R4.2.1 and SPSS 20.0 (IBM, Chicago, United States). Univariate ANOVA (Tukey’s HSD, *p* < 0.05) was used to analyze significant differences in soil chemical and physical properties and abundance of functional genes during thawing. The Mantel test established through the “linkET” program package in R language was used to analyze the interaction influences of different factors. Partial least squares path modeling (PLS-PM) was established through the “plsm” program package in R language to analyze the influence of biochar addition and moisture on N_2_O emissions during the thawing process.

## Results

### Soil N_2_O emissions

**Figure 2:**
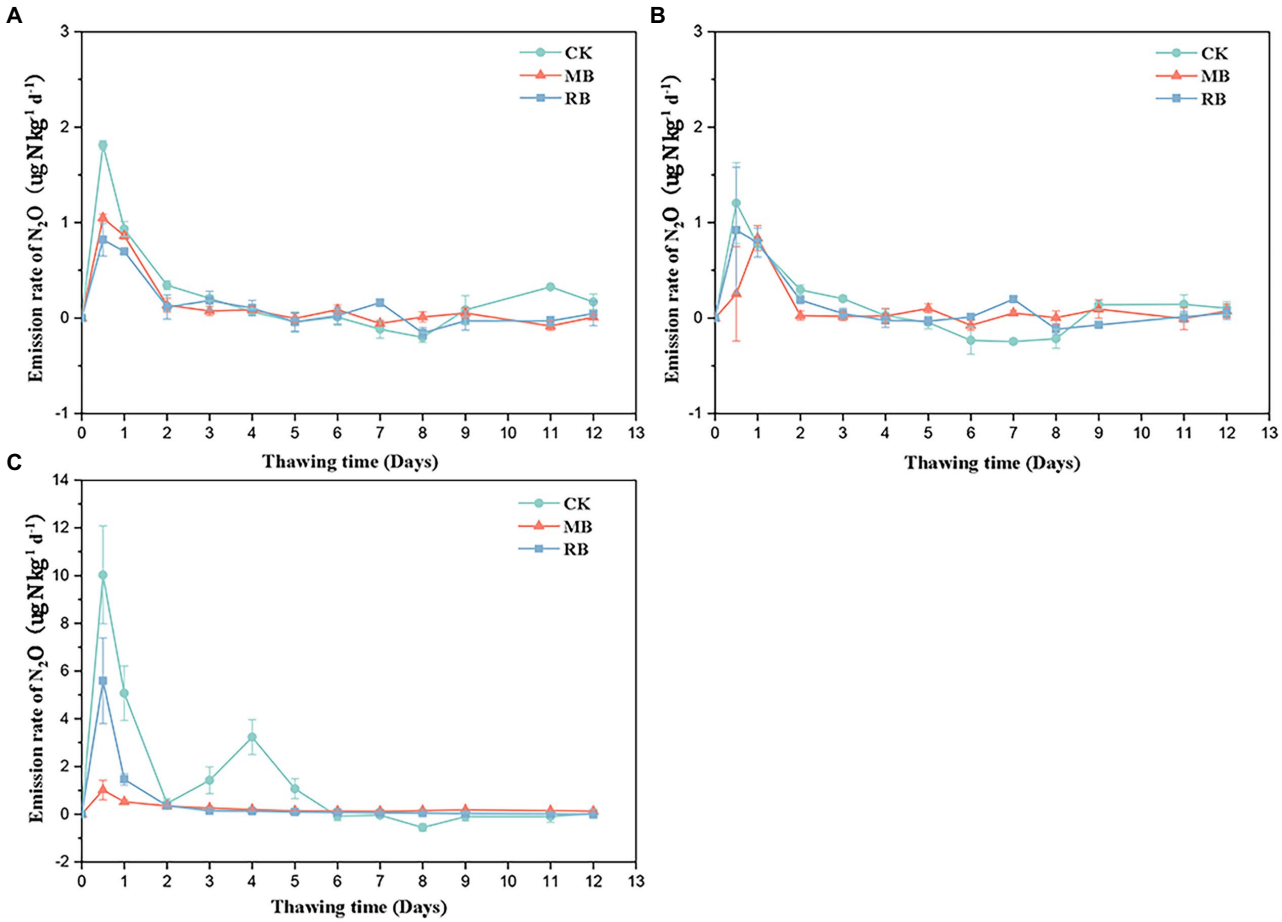
Emission rates of soil nitrous oxide (N_2_O) with biochar amendments during the thawing period under 60% water holding capacity (WHC; **A**), 100% WHC **(B)**, and flooding **(C)**. (MB: maize straw biochar; RB: rice straw biochar; error bars represent the standard error, *n* = 3).

**Figure 3:**
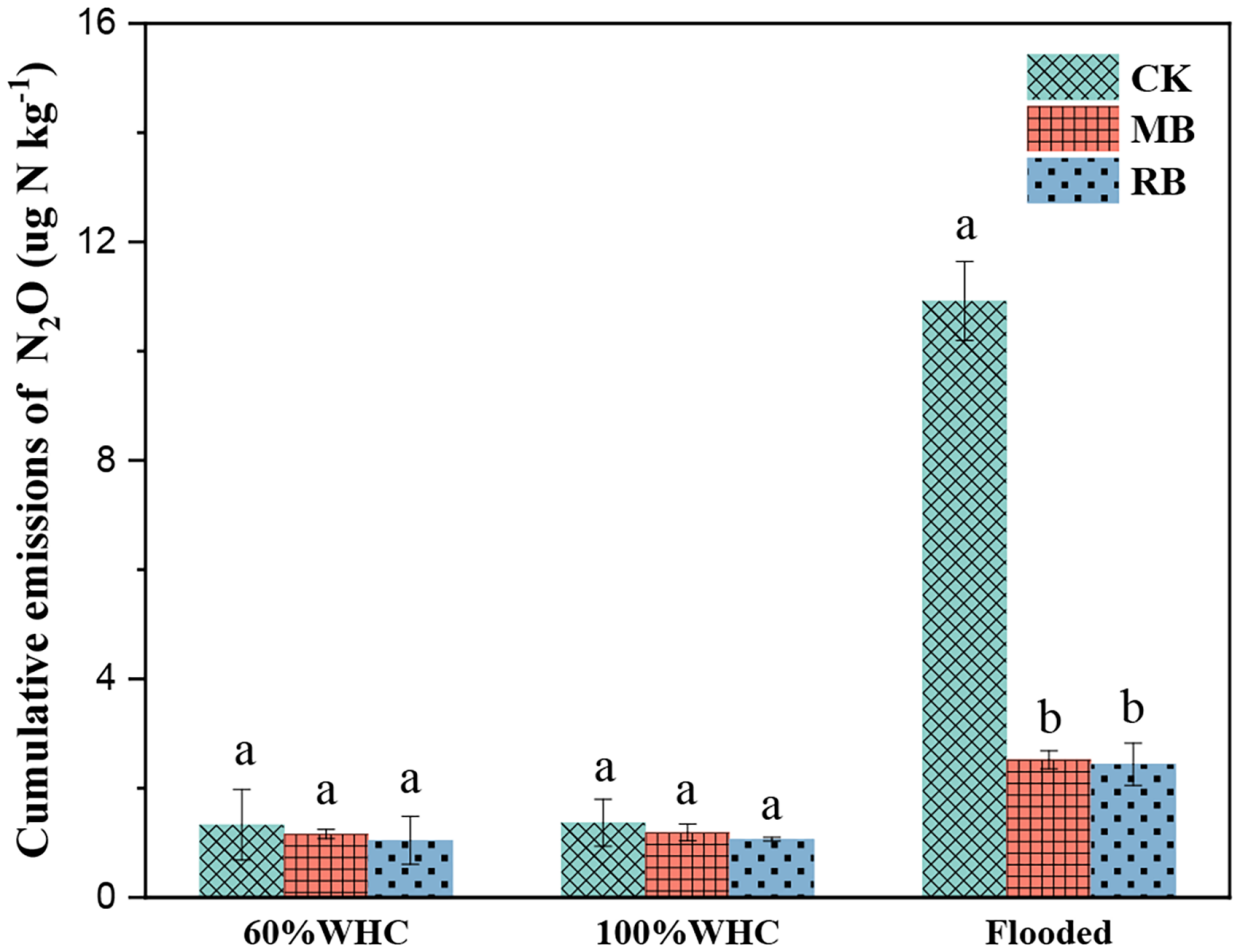
Cumulative N_2_O emissions with biochar amendments during the thawing period. (CK: control; MB: maize straw biochar; RB: rice straw biochar; and a and b indicate significant differences among the different biochar treatments, *p* < 0.05; error bars represent the standard error, *n* = 3).

After different biochar treatments, N_2_O gas emissions peaked at the initial stage of soil thawing (within 1 day), declined rapidly, and then tended to be stable (Figure 2). The addition of both biochar types had a remarkable influence on cumulative N_2_O emissions during soil thawing in flooded conditions, while there was no remarkable difference in total N_2_O emissions between the two nonflooded conditions (Figure 3). The MB and RB treatments inhibited cumulative N_2_O emissions by 67 and 69%, respectively. No significant difference was found between the inhibition effects of these two biochars on N_2_O emissions during the soil freeze–thaw process.

### Soil mineral N and DOC

**Figure 4:**
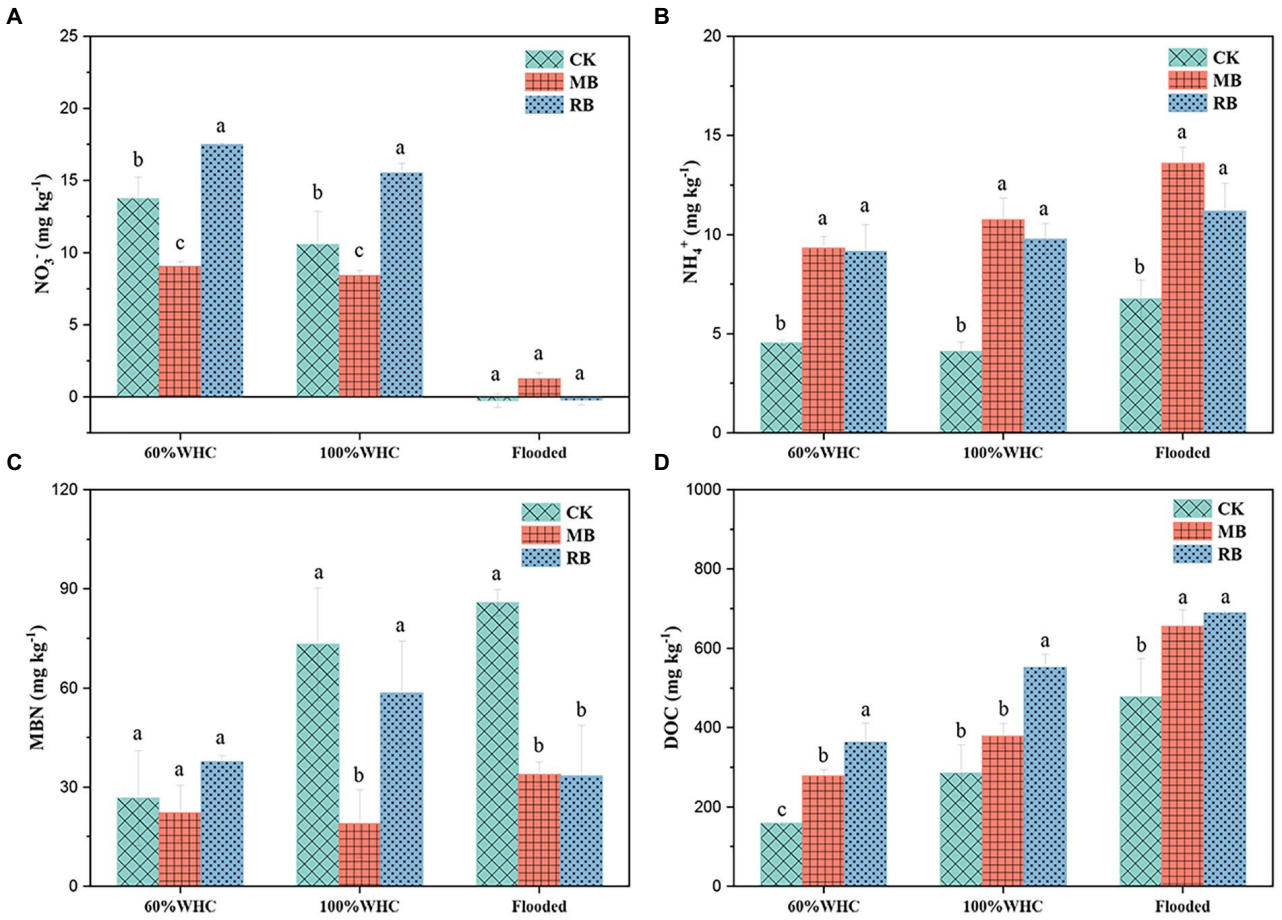
Contents of soil NO_3_^−^ **(A)**, NH_4_^+^ **(B)**, microbial biomass nitrogen (MBN; **C**), and dissolved organic carbon (DOC; **D**) with biochar amendments on the first day after thawing. (CK: control; MB: maize straw biochar; RB: rice straw biochar; a and b indicate significant differences among the different biochar treatments, *p* < 0.05; error bars represent the standard error, *n* = 3).

In our study, contrasted with the control treatment, the NO_3_^−^ content in the nonflooded soil under the MB treatment decreased at 1 day of thawing, while the NO_3_^−^ content in the nonflooded soil increased under the RB treatment. There was no significant difference in NO_3_^−^ content between the different treatments under flooded conditions (Figure 4A). Both biochar amendments increased NH_4_^+^ content, but there was no significant difference in NH_4_^+^ content between the two treatments (Figure 4B). Compared with the control treatment, biochar addition increased the content of DOC by 0.32–1.55 times. Moreover, the soil DOC content with RB addition was higher than that with MB addition (Figure 4D). Last, biochar addition reduced the soil MBN content under 100% WHC and flooded soils but did not affect MBN content under 60% WHC soil (Figure 4C).

### Microbial functional genes

**Figure 5:**
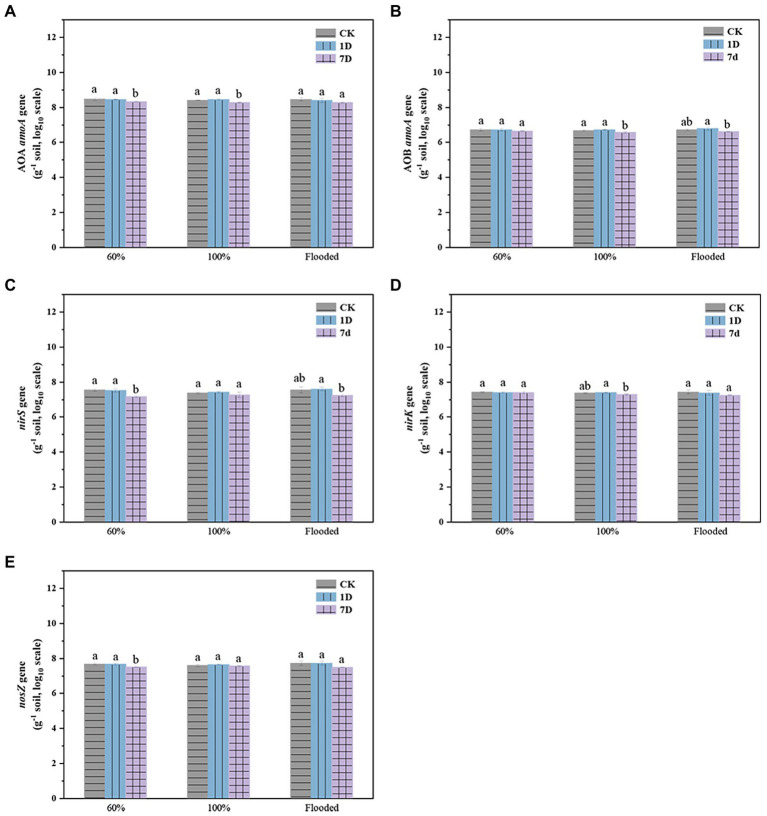
Abundance of the AOA *amoA* **(A)**, AOB *amoA* **(B)**, *nirS* **(C)**, *nirK* **(D)**, and *nosZ* **(E)** genes at different times (CK: control; 1D: the first day after thawing; 7D: the seventh day after thawing; a and b indicate significant differences among the different period, *p* < 0.05; error bars represent the standard error, *n* = 3).

**Figure 6:**
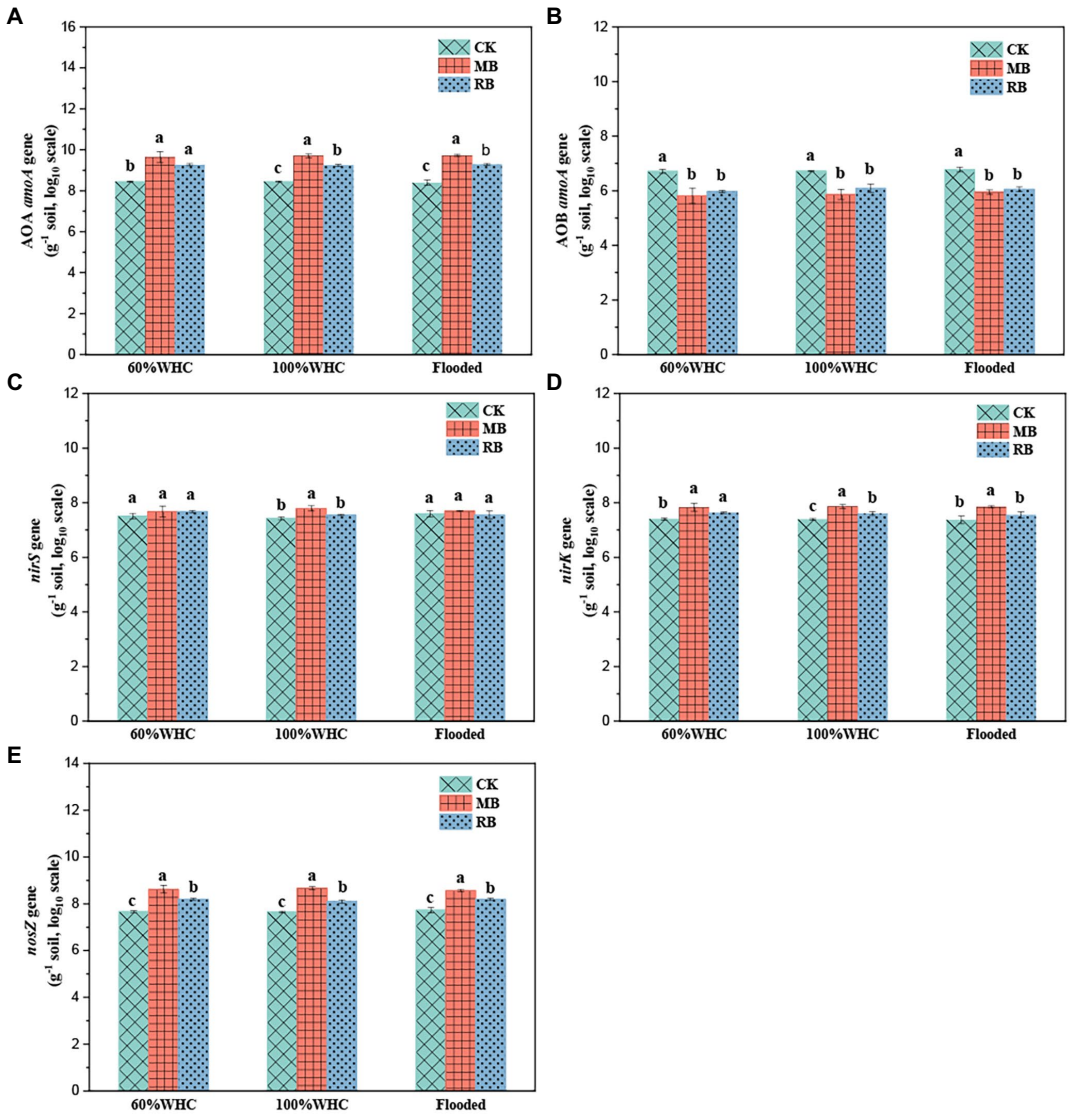
Abundance of the AOA *amoA* **(A)**, AOB *amoA* **(B)**, *nirS* **(C)**, *nirK* **(D)**, and *nosZ* **(E)** genes with biochar amendments on the first day after soil thawing (CK: control; MB: maize straw biochar; RB: rice straw biochar; a, b and c indicate significant differences among the different biochar treatments, *p* < 0.05; error bars represent the standard error, *n* = 3).

**Figure 7:**
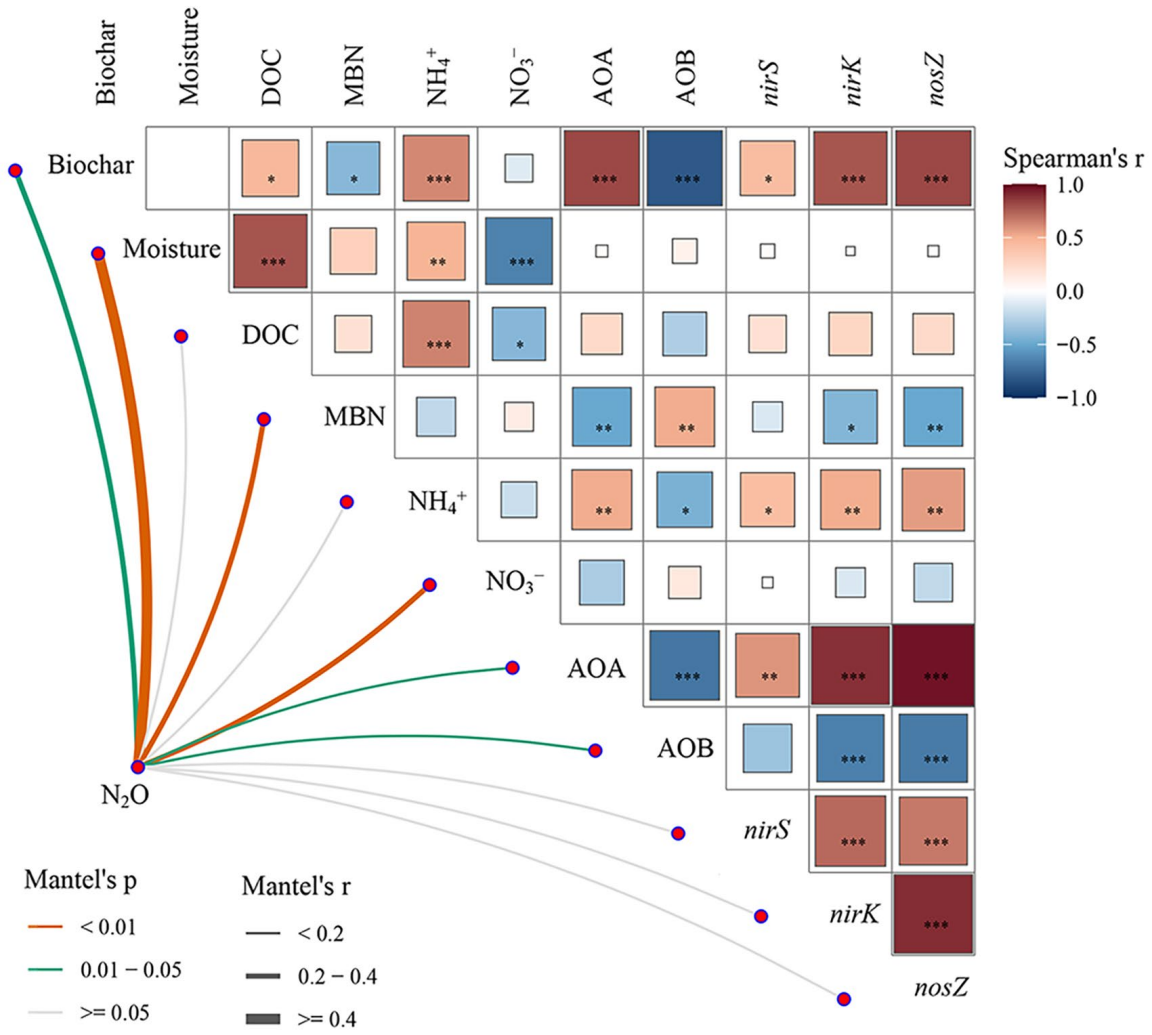
Pairwise comparisons of different factors are shown, with a color gradient denoting Spearman’s correlation coefficients. N_2_O emissions were related to each factor by Mantel tests. Edge width corresponds to Mantel’s r statistic for the corresponding distance correlations, and edge color denotes the statistical significance based on 9,999 permutations. *, **, and *** indicate *p* < 0.05, *p* < 0.01, and *p* < 0.001, respectively.

**Figure 8:**
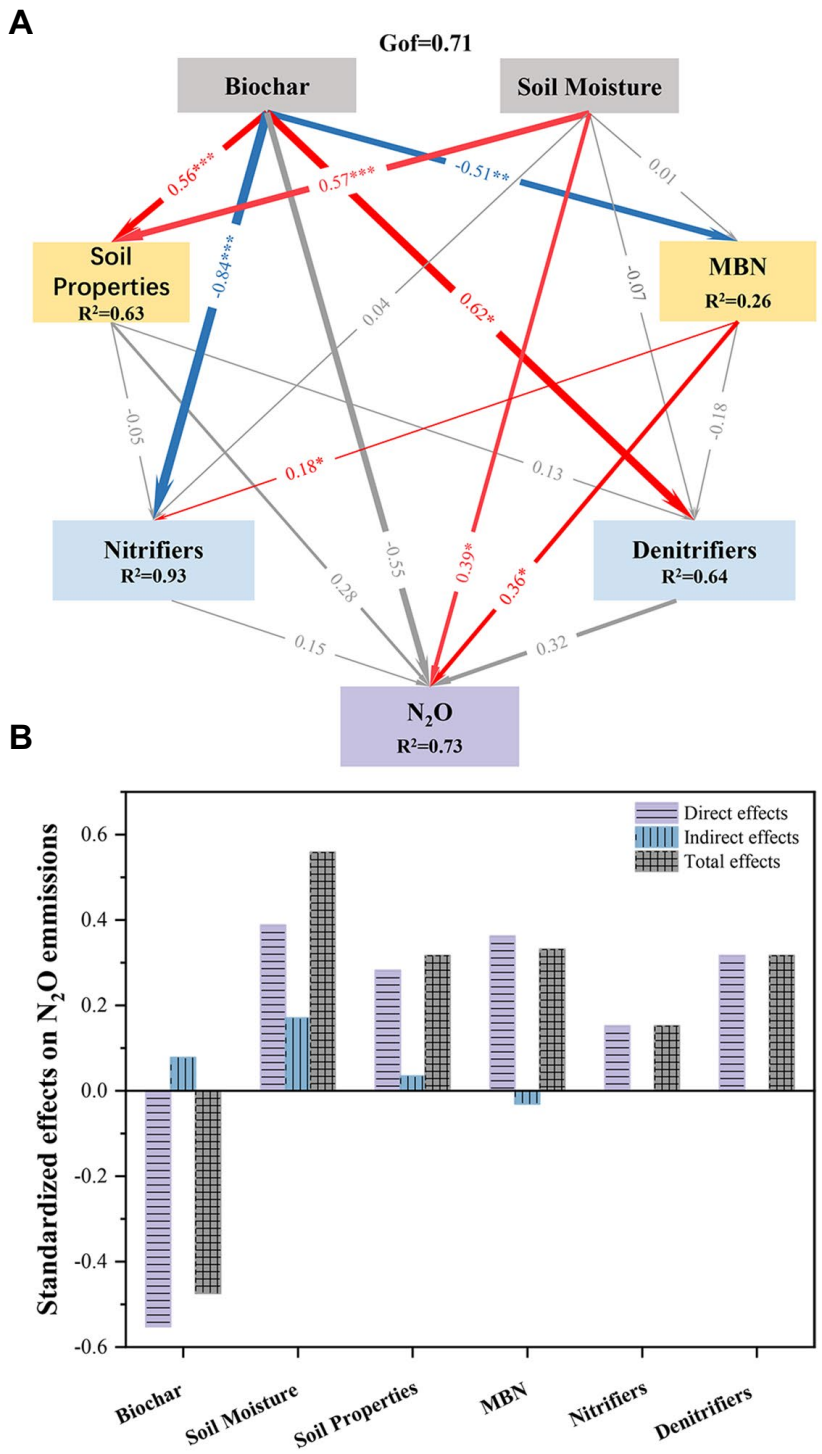
PLS-PM showing the direct and indirect effects of different factors on N_2_O emissions with biochar amendment. (**A**: PLS-PM showing the relationships among biochar, soil moisture, soil properties, microbial biomass nitrogen, nitrifiers, and denitrifiers with respect to N_2_O emissions. Blue and red arrows indicate positive and negative effects, respectively; the width of arrows indicates the path coefficients; *, **, and *** indicate *p* < 0.05, *p* < 0.01, and *p* < 0.001, respectively; Gof indicates the goodness of fit; **B**: Standardized direct and indirect mean effects).

There was no significant difference in functional gene abundance related to nitrification and denitrification processes on the first day after thawing compared with before freezing, but most of the gene abundance decreased on the seventh day after thawing (Figure 5). The addition of biochar increased the abundance of the nitrifying gene AOA *amoA* (Figure 6A) but reduced AOB *amoA* (Figure 6B). Biochar treatment did not observably influence the abundance of the denitrifying functional gene *nirS* (Figure 6C) but increased the abundance of *nirK* (Figure 6D) and *nosZ* (Figure 6E). In addition, soil moisture had no significant effect on functional gene abundance (Figures 7, 8).

### Relationship among different factors and N_2_O emissions

The Mantel test showed that biochar, soil moisture, MBN, NO_3_^−^, AOA *amoA*, and AOB *amoA* had significant influences on N_2_O emissions. Moreover, soil moisture had negative effects on the content of NO_3_^−^ (Figure 7). PLS-PM showed that the addition of biochar had significant negative effects on nitrifiers and MBN content and indirectly affected N_2_O emissions through its effect on soil MBN (Figures 8A,B). Soil moisture directly significantly affects N_2_O emissions and indirectly affects N_2_O emissions through its influence on soil physicochemical properties (Figures 8A,B).

## Discussion

### Effect of soil moisture on N_2_O emissions during thawing

Previous studies observed peaks of N_2_O emission at the start of thawing during the freeze–thaw periods (Prieme and Christensen, 2001; Wu et al., 2020). In the present study, N_2_O gas emissions peaked at the initial stage of soil thawing, which mainly contributed to the release of N_2_O during the freeze–thaw period. There was no significant difference in N_2_O emissions (< 2 μg kg^−1^) found in this study between soil moisture contents of 60 and 100% WHC during the freeze–thaw process, but the cumulative N_2_O emissions significantly increased to 10.9 μg kg^−1^ in the flooded soil. This result was consistent with Bhowmik et al. (2016), who found that N_2_O emissions from pasture soil increased from 5.43 to 12.3 μg kg^−1^ during freeze–thaw when the soil moisture content increased from 40 to 80%. This result was probably due to denitrification dominating the N_2_O production under saturated water content and then inducing a high risk of N_2_O emission during the thawing process (Mathieu et al., 2006; Braker et al., 2010). Overall, our results highlight the importance of water conditions in regulating N_2_O emissions during thawing, and it is necessary to develop an efficient approach to inhibit the production of N_2_O under flooded conditions.

Soil moisture controls the emission of N_2_O mainly by regulating the content of soil oxygen (O_2_) and thereby affects the transformation of denitrification and nitrification processes (Li et al., 2021). The flooding conditions with high water content in soil reduced O_2_ diffusion and formed anoxic or anaerobic environments, which are conducive to denitrification (Ma and Fan, 2020). However, in this study, there was no significant difference in the abundance of functional genes related to nitrification and denitrification processes. But the soil NO_3_^−^ content, as a substrate for denitrifiers, decreased sharply under flooding conditions during thawing. This result showed that soil moisture probably affects N_2_O production/reduction by controlling denitrifying microbial activity rather than denitrifying microbial gene abundance.

### Effect of biochar addition on N_2_O emissions during thawing

Previous studies found that biochar can effectively reduce soil greenhouse gas production (Yin et al., 2021), which might be a possible method to decrease soil N_2_O emissions during freeze–thaw. Our results demonstrated that biochar amendment significantly inhibited soil N_2_O production by 67–69% during freeze–thaw, which was consistent with the results of Liu et al. (2016), who confirmed that biochar addition inhibited soil N_2_O emissions by 20–70% during freeze–thaw. In another experiment, Hou et al. (2020) found that the application of biochar to seasonally frozen farmland soil directly reduced cumulative N_2_O emissions by 24%. However, Zhou et al. (2017) found that biochar with high porosity interacted with freezing and increased N_2_O emissions by microbial lysis. This difference indicated that the effect of biochar amendments on N_2_O emissions during the freeze–thaw process varied with their type. Moreover, based on a meta-analysis, Brassard et al. (2016) found that biochar with a lower nitrogen content and higher C/N ratio (>30) was more suitable for mitigating soil N_2_O emissions. In this study, compared with MB, although RB contained lower nitrogen content but higher porosity, there was no significant difference in N_2_O mitigation potential between the two treatments amended with MB or RB biochar under flooded conditions. This result indicates that the anaerobic environment probably masked the actual impact of biochar properties on N_2_O emissions.

It has been reported that biochar can reduce N_2_O emissions by affecting soil N availability (Liu et al., 2016). However, under near-saturated conditions, Case et al. (2015) found that biochar inhibited the release of N_2_O emissions, but the change in soil inorganic nitrogen (including NH_4_^+^ and NO_3_^¯^) was not able to explain the reduction in N_2_O emissions. In this study, the amendment of rice straw biochar significantly increased NH_4_^+^ and NO_3_^¯^ contents but had a negligible effect on N_2_O emissions, indicating that biochar amendment did not affect inorganic N availability for N_2_O production (Case et al., 2015; Xie et al., 2020). Moreover, under flooded conditions, the amendment of biochar increased the NH_4_^+^ content, which supplied an N source for nitrification to produce N_2_O. However, the application of biochar significantly reduced N_2_O emissions, indicating that the reduction in N_2_O was probably due to the inhibitory compounds of biochar on nitrifiers and denitrifiers (Taghizadeh-Toosi et al., 2011; Quilliam et al., 2013; Case et al., 2015) rather than the N substrates.

The inhibitory effect of biochar on N_2_O formation was mainly due to its inhibitory compounds on denitrification (Case et al., 2015; Edwards et al., 2018). However, in this study, biochar amendments significantly increased the abundance of the AOA *amoA* gene but reduced AOB *amoA* gene abundance during the freeze–thaw period. In neutral and alkaline soils, Shen et al. (2012) found that AOB dominate nitrification rather than AOA using the DNA stable isotope probing (DNA-SIP) method. Therefore, it is expected that biochar can reduce N_2_O production, probably by limiting the nitrification caused by AOB. During the denitrification process, the amendment of biochar significantly increased the abundance of the *nosZ* gene encoding N_2_O reductase, indicating that biochar reduced N_2_O emission through enhanced N_2_O reduction during thawing. In addition, the addition of biochar significantly decreased the content of MBN, indicating that biochar reduced N_2_O emissions by inhibiting the substrates for N mineralization. Overall, based on the PLS-PM model, although biochar significantly affects the abundance of nitrifiers and denitrifiers, the change in functional genes related to nitrification and denitrification was not able to explain the reduction in N_2_O emissions. Further study should focus on the impact of biochar on the activity of nitrifiers and denitrifiers.

## Conclusion

In the absence of biochar, there was no significant difference in N_2_O emissions between soil water holding capacities of 60 and 100% during the freezing–thawing period. However, microbial-mediated denitrification and nitrification processes in flooded soil led to a large increase in N_2_O produced by freeze–thaw processes. During the thawing period, the application of biochar to soil had a negligible effect on N_2_O emissions under nonflooded conditions but significantly reduced cumulative N_2_O emissions by 67–69% under flooded condition. The reduction in N_2_O emissions in the flooded environment was mainly due to biochar reducing N_2_O production by decreasing the AOB *amoA* gene abundance and enhancing N_2_O reduction by increasing the *nosZ* gene, which encodes N_2_O reductase. However, across the different water conditions, the change in functional genes related to nitrification and denitrification processes was not able to explain the reduction in N_2_O emissions. Further study should focus on the impact of biochar on the activity of nitrifiers and denitrifiers. Overall, our study showed that field water management was important for the release of N_2_O in the freezing–thawing stage of paddy soil, and biochar addition could alleviate the N_2_O produced in the freezing–thawing stage of flooding soil.

## Data availability statement

The raw data supporting the conclusions of this article will be made available by the authors, without undue reservation.

## Author contributions

HY and YY: conceptualization, supervision, and funding acquisition. HY, YY, RS, and ZY: methodology. ZY, RS, and YY: software, formal analysis, and data curation. RS, YY, LH, and ZY: validation. RS and LH: investigation. YY and HY: resources and visualization. ZY, RS, and LH: writing–original draft preparation. LH and YY: writing–review and editing. YY and RS: project administration. All authors contributed to the article and approved the submitted version.

## Funding

This work was funded by the National Natural Science Foundation of China (42277109, 42077036, and 42021005) and the Ningbo Key Research and Development Program (2022Z159).

## Conflict of interest

The authors declare that the research was conducted in the absence of any commercial or financial relationships that could be construed as a potential conflict of interest.

## Publisher’s note

All claims expressed in this article are solely those of the authors and do not necessarily represent those of their affiliated organizations, or those of the publisher, the editors and the reviewers. Any product that may be evaluated in this article, or claim that may be made by its manufacturer, is not guaranteed or endorsed by the publisher.

## References

[ref1] Ahmad Z. Mosa A. Zhan L. Gao B. (2021). Biochar modulates mineral nitrogen dynamics in soil and terrestrial ecosystems: a critical review. Chemosphere 278:130378. doi: 10.1016/j.chemosphere.2021.130378, PMID: 33838428

[ref2] Bhowmik A. Fortuna A. M. Cihacek L. J. Bary A. I. Cogger C. G. (2016). Use of biological indicators of soil health to estimate reactive nitrogen dynamics in long-term organic vegetable and pasture systems. Soil Biol. Biochem. 103, 308–319. doi: 10.1016/j.soilbio.2016.09.004

[ref3] Braker G. Schwarz J. Conrad R. (2010). Influence of temperature on the composition and activity of denitrifying soil communities. FEMS Microbiol. Ecol. 73, 134–148. doi: 10.1111/j.1574-6941.2010.00884.x, PMID: 20455938

[ref4] Brassard P. Godbout S. Raghavan V. (2016). Soil biochar amendment as a climate change mitigation tool: key parameters and mechanisms involved. J. Environ. Manag. 181, 484–497. doi: 10.1016/j.jenvman.2016.06.063, PMID: 27420171

[ref5] Bruun E. W. Muller-Stover D. Ambus P. Hauggaard-Nielsen H. (2011). Application of biochar to soil and N_2_O emissions: potential effects of blending fast-pyrolysis biochar with anaerobically digested slurry. Eur. J. Soil Sci. 62, 581–589. doi: 10.1111/j.1365-2389.2011.01377.x

[ref6] Case S. D. C. McNamara N. P. Reay D. S. Stott A. W. Grant H. K. Whitaker J. (2015). Biochar suppresses N_2_O emissions while maintaining N availability in a sandy loam soil. Soil Biol. Biochem. 81, 178–185. doi: 10.1016/j.soilbio.2014.11.012

[ref7] Chen Z. Yang S. Q. Zhang A. P. Jing X. Song W. M. Mi Z. R. (2018). Nitrous oxide emissions following seasonal freeze-thaw events from arable soils in Northeast China. J. Integr. Agric. 17, 231–246. doi: 10.1016/s2095-3119(17)61738-6

[ref8] Clough T. J. Bertram J. E. Ray J. L. Condron L. M. O’Callaghan M. Sherlock R. R. (2010). Unweathered wood biochar impact on nitrous oxide emissions from a bovine-urine-amended pasture soil. Soil Sci. Soc. Am. J. 74, 852–860. doi: 10.2136/sssaj2009.0185

[ref9] Edwards J. D. Pittelkow C. M. Kent A. D. Yang W. H. (2018). Dynamic biochar effects on soil nitrous oxide emissions and underlying microbial processes during the maize growing season. Soil Biol. Biochem. 122, 81–90. doi: 10.1016/j.soilbio.2018.04.008

[ref10] Francis C. A. Roberts K. J. Beman J. M. Santoro A. E. Oakley B. B. (2005). Ubiquity and diversity of ammonia-oxidizing archaea in water columns and sediments of the ocean. Proc. Natl. Acad. Sci. U. S. A. 102, 14683–14688. doi: 10.1073/pnas.0506625102, PMID: 16186488 PMC1253578

[ref11] Gao Y. H. Zeng X. Y. Xie Q. Y. Ma X. X. (2015). Release of carbon and nitrogen from alpine soils during thawing periods in the eastern Qinghai-Tibet plateau. Water Air Soil Pollut. 226:209. doi: 10.1007/s11270-015-2479-2

[ref12] Goldberg S. D. Borken W. Gebauer G. (2010). N_2_O emission in a Norway spruce forest due to soil frost: concentration and isotope profiles shed a new light on an old story. Biogeochemistry 97, 21–30. doi: 10.1007/s10533-009-9294-z

[ref13] Hou R. J. Li T. X. Fu Q. Liu D. Li M. Zhou Z. Q. (2020). Effects of biochar and straw on greenhouse gas emission and its response mechanism in seasonally frozen farmland ecosystems. Catena 194:104735. doi: 10.1016/j.catena.2020.104735

[ref14] Jenkinson D. S. Brookes P. C. Powlson D. S. (2004). Measuring soil microbial biomass. Soil Biol. Biochem. 36, 5–7. doi: 10.1016/j.soilbio.2003.10.002

[ref15] Kim D. G. Vargas R. Bond-Lamberty B. Turetsky M. R. (2012). Effects of soil rewetting and thawing on soil gas fluxes: a review of current literature and suggestions for future research. Biogeosciences 9, 2459–2483. doi: 10.5194/bg-9-2459-2012

[ref16] Li J. B. Zhao Y. Zhang A. F. Song B. Hill R. L. (2021). Effect of grazing exclusion on nitrous oxide emissions during freeze-thaw cycles in a typical steppe of Inner Mongolia. Agric. Ecosyst. Environ. 307:107217. doi: 10.1016/j.agee.2020.107217

[ref17] Liu X. Wang Q. Qi Z. M. Han J. G. Li L. H. (2016). Response of N_2_O emissions to biochar amendment in a cultivated sandy loam soil during freeze-thaw cycles. Sci. Rep. 6:35411. doi: 10.1038/srep35411, PMID: 27748462 PMC5066323

[ref18] Ma Y. H. Fan X. J. (2020). Detection and analysis of soil water content based on experimental reflectance spectrum data. Asia Pac. J. Chem. Eng. 15:e2507. doi: 10.1002/apj.2507

[ref19] Mathieu O. Henault C. Leveque J. Baujard E. Milloux M. J. Andreux F. (2006). Quantifying the contribution of nitrification and denitrification to the nitrous oxide flux using N-15 tracers. Environ. Pollut. 144, 933–940. doi: 10.1016/j.envpol.2006.02.005, PMID: 16569469

[ref20] Mellander P. E. Lofvenius M. O. Laudon H. (2007). Climate change impact on snow and soil temperature in boreal scots pine stands. Clim. Chang. 85, 179–193. doi: 10.1007/s10584-007-9254-3

[ref21] Pelster D. E. Chantigny M. H. Rochette P. Bertrand N. Angers D. Zebarth B. J. (2019). Rates and intensity of freeze-thaw cycles affect nitrous oxide and carbon dioxide emissions from agricultural soils. Can. J. Soil Sci. 99, 472–484. doi: 10.1139/cjss-2019-0058

[ref22] Peng B. Sun J. F. Liu J. Dai W. W. Sun L. F. Pei G. T. (2019). N_2_O emission from a temperate forest soil during the freeze-thaw period: a mesocosm study. Sci. Total Environ. 648, 350–357. doi: 10.1016/j.scitotenv.2018.08.155, PMID: 30121034

[ref23] Prieme A. Christensen S. (2001). Natural perturbations, drying-wetting and freezing-thawing cycles, and the emission of nitrous oxide, carbon dioxide and methane from farmed organic soils. Soil Biol. Biochem. 33, 2083–2091. doi: 10.1016/s0038-0717(01)00140-7

[ref24] Purakayastha T. J. Bera T. Bhaduri D. Sarkar B. Mandal S. Wade P. (2019). A review on biochar modulated soil condition improvements and nutrient dynamics concerning crop yields: pathways to climate change mitigation and global food security. Chemosphere 227, 345–365. doi: 10.1016/j.chemosphere.2019.03.170, PMID: 30999175

[ref25] Quilliam R. S. Rangecroft S. Emmett B. A. Deluca T. H. Jones D. L. (2013). Is biochar a source or sink for polycyclic aromatic hydrocarbon (PAH) compounds in agricultural soils? Glob. Change Biol. Bioenergy 5, 96–103. doi: 10.1111/gcbb.12007

[ref26] Scala D. J. Kerkhof L. J. (1998). Nitrous oxide reductase (nos Z) gene-specific PCR primers for detection of denitrifiers and three *nos Z* genes from marine sediments. FEMS Microbiol. Lett. 162, 61–68. doi: 10.1016/s0378-1097(98)00103-7, PMID: 9595664

[ref27] Scheer C. Grace P. R. Rowlings D. W. Kimber S. Van Zwieten L. (2011). Effect of biochar amendment on the soil-atmosphere exchange of greenhouse gases from an intensive subtropical pasture in northern New South Wales. Plant Soil 345, 47–58. doi: 10.1007/s11104-011-0759-1

[ref28] Shen J. P. Zhang L. M. Di H. J. He J. Z. (2012). A review of ammonia-oxidizing bacteria and archaea in Chinese soils. Front. Microbiol. 3:296. doi: 10.3389/fmicb.2012.00296, PMID: 22936929 PMC3424668

[ref29] Song X. Y. Wang G. X. Ran F. Huang K. W. Sun J. Y. Song C. L. (2020). Soil moisture as a key factor in carbon release from thawing permafrost in a boreal forest. Geoderma 357:113975. doi: 10.1016/j.geoderma.2019.113975

[ref30] Song C. C. Zhang J. B. Wang Y. Y. Wang Y. S. Zhao Z. C. (2008). Emission of CO_2_, CH_4_ and N_2_O from freshwater marsh in northeast of China. J. Environ. Manag. 88, 428–436. doi: 10.1016/j.jenvman.2007.03.030, PMID: 17517465

[ref31] Spokas K. A. Novak J. M. Stewart C. E. Cantrell K. B. Uchimiya M. DuSaire M. G. (2011). Qualitative analysis of volatile organic compounds on biochar. Chemosphere 85, 869–882. doi: 10.1016/j.chemosphere.2011.06.108, PMID: 21788060

[ref32] Stres B. Danevcic T. Pal L. Fuka M. M. Resman L. Leskovec S. (2008). Influence of temperature and soil water content on bacterial, archaeal and denitrifying microbial communities in drained fen grassland soil microcosms. FEMS Microbiol. Ecol. 66, 110–122. doi: 10.1111/j.1574-6941.2008.00555.x, PMID: 18710395

[ref33] Taghizadeh-Toosi A. Clough T. J. Condron L. M. Sherlock R. R. Anderson C. R. Craigie R. A. (2011). Biochar incorporation into pasture soil suppresses in situ nitrous oxide emissions from ruminant urine patches. J. Environ. Qual. 40, 468–476. doi: 10.2134/jeq2010.0419, PMID: 21520754

[ref34] Throback I. N. Enwall K. Jarvis A. Hallin S. (2004). Reassessing PCR primers targeting nirS, nirK and *nosZ* genes for community surveys of denitrifying bacteria with DGGE. FEMS Microbiol. Ecol. 49, 401–417. doi: 10.1016/j.femsec.2004.04.011, PMID: 19712290

[ref35] Wagner-Riddle C. Congreves K. A. Abalos D. Berg A. A. Brown S. E. Ambadan J. T. (2017). Globally important nitrous oxide emissions from croplands induced by freeze-thaw cycles. Nat. Geosci. 10:279. doi: 10.1038/ngeo2907

[ref36] Wu X. Li T. Wang D. B. Wang F. F. Fu B. J. Liu G. H. (2020). Soil properties mediate the freeze-thaw-related soil N_2_O and CO_2_ emissions from temperate grasslands. Catena 195:104797. doi: 10.1016/j.catena.2020.104797

[ref37] Xie Y. Yang C. Ma E. D. Tan H. Zhu T. B. Muller C. (2020). Biochar stimulates NH_4_^+^ turnover while decreasing NO_3_^−^ production and N_2_O emissions in soils under long-term vegetable cultivation. Sci. Total Environ. 737:140266. doi: 10.1016/j.scitotenv.2020.140266, PMID: 32783855

[ref38] Yao Z. S. Wu X. Wolf B. Dannenmann M. Butterbach-Bahl K. Brueggemann N. (2010). Soil-atmosphere exchange potential of NO and N_2_O in different land use types of Inner Mongolia as affected by soil temperature, soil moisture, freeze-thaw, and drying-wetting events. J. Geophys. Res.-Atmos. 115:D17116. doi: 10.1029/2009jd013528

[ref39] Yin M. Gao X. Tenuta M. Gui D. Zeng F. (2019). Presence of spring-thaw N_2_O emissions are not linked to functional gene abundance in a drip-fertigated cropped soil in arid northwestern China. Sci. Total Environ. 695:133670. doi: 10.1016/j.scitotenv.2019.133670, PMID: 31412304

[ref40] Yin Y. A. Yang C. Li M. T. Zheng Y. C. Ge C. J. Gu J. (2021). Research progress and prospects for using biochar to mitigate greenhouse gas emissions during composting: a review. Sci. Total Environ. 798:149294. doi: 10.1016/j.scitotenv.2021.149294, PMID: 34332388

[ref41] Yu Y. X. Li X. Feng Z. Y. Xiao M. L. Ge T. D. Li Y. Y. (2022). Polyethylene microplastics alter the microbial functional gene abundances and increase nitrous oxide emissions from paddy soils. J. Hazard. Mater. 432:128721. doi: 10.1016/j.jhazmat.2022.128721, PMID: 35334262

[ref42] Zhang Y. X. Li X. Xiao M. Feng Z. Y. Yu Y. X. Yao H. Y. (2022b). Effects of microplastics on soil carbon dioxide emissions and the microbial functional genes involved in organic carbon decomposition in agricultural soil. Sci. Total Environ. 806:150714. doi: 10.1016/j.scitotenv.2021.150714, PMID: 34606872

[ref43] Zhang L. Y. Zhang M. X. Li Y. T. Li J. L. Jing Y. M. Xiang Y. Z. (2022a). Linkage of crop productivity to soil nitrogen dynamics under biochar addition: a meta-analysis across field studies. Agronomy 12:247. doi: 10.3390/agronomy12020247

[ref44] Zhang B. Zhou M. Zhu B. Xiao Q. Wang T. Tang J. (2021). Soil type affects not only magnitude but also thermal sensitivity of N_2_O emissions in subtropical mountain area. Sci. Total Environ. 797:149127. doi: 10.1016/j.scitotenv.2021.149127 34311350

[ref45] Zhou Y. X. Berruti F. Greenhalf C. Tian X. H. Henry H. A. L. (2017). Increased retention of soil nitrogen over winter by biochar application: implications of biochar pyrolysis temperature for plant nitrogen availability. Agric. Ecosyst. Environ. 236, 61–68. doi: 10.1016/j.agee.2016.11.011

